# Utilizing environmental DNA and imaging to study the deep-sea fish community of Takuyo-Daigo Seamount

**DOI:** 10.1038/s44185-024-00042-w

**Published:** 2024-05-31

**Authors:** Akira Iguchi, Miyuki Nishijima, Eri Ikeuchi, Hiroyuki Yokooka, Hideki Sugishima, Kazumasa Ikeda, Ryuichi Miwa, Yoshiro Sekido, Nozomu Iwasaki, Masahiro Suzumura, Ayumi Tsukasaki, Yuichiro Tanaka, Shogo Kato, Jumpei Minatoya, Nobuyuki Okamoto, Taiga Kunishima, Yuji Ise, Atsushi Suzuki

**Affiliations:** 1 Geological Survey of Japan, National Institute of Advanced Industrial Science and Technology (AIST), 1-1-1 Higashi, Tsukuba, Ibaraki 305-8567 Japan; 2 Research Laboratory on Environmentally-conscious Developments and Technologies [E-code], National Institute of Advanced Industrial Science and Technology (AIST), Tsukuba, 305-8567 Japan; 3 IDEA Consultants, Inc., 1334-5 Riemon, Yaizu, Shizuoka 421-0212 Japan; 4 Okamoto Glass Co., Ltd., 380 Toyofuta, Kashiwa, Chiba 277-0872 Japan; 5 Kaiyo Engineering Co., Ltd., 4-28-11 Taito, Taito, Tokyo 110-0016 Japan; 6 Marine Biological Research Institute of Japan Co., Ltd., 4-28-11 Taito, Taito, Tokyo 110-0016 Japan; 7 Faculty of Geo-Environmental Science, Rissho University, 1700 Magechi, Kumagaya, Saitama 360-0194 Japan; 8 Environmental Management Research Institute, National Institute of Advanced Industrial Science and Technology (AIST), 16-1 Onogawa, Tsukuba, Ibaraki 305-8569 Japan; 9 Deep Ocean Resources Development CO., Ltd., 2-3-5, Nihonbashi Horidome-cho, Chuoh-ku, Tokyo 103-0012 Japan; 10 Faculty of Agriculture, Setsunan University, 45-1 Nagaotoge-cho, Hirakata, Osaka 573-0101 Japan; 11 Kuroshio Biological Research Foundation, 560 Nishidomari, Otsuki, Kochi 788-0333 Japan

**Keywords:** Biodiversity, Conservation biology

## Abstract

The increase in interest of mining at seamounts means there is a critical need to establish baseline inventories through environmental survey, with the aim of promoting the conservation and stewardship of these remote habitats. To efficiently evaluate fish biodiversity around a seamount, we compared environmental DNA (eDNA) methods using seawater and sponge samples against methods using imagery obtained with a remotely operated vehicle (ROV) and a free-fall deep-sea camera lander called the Edokko Mark I on the Takuyo-Daigo Seamount (153.0°E, 23.5°N) in the northwestern Pacific Ocean. We detected a total of 18 fish families by these methods. The fish fauna detected on the seamount included many families commonly found in deep-sea areas and were similar to the fish fauna of other seamounts located at similar latitudes in the northwestern Pacific. Significant differences in the patterns of detection of fish families between the eDNA and imaging methods is attributed to the differing powers of detection of some fish groups between methods (related to primer compatibility and fish size). For deep-sea fish, the difference in fish composition at the family level between seawater and sponge eDNA methods was not significant, but the difference between Edokko Mark I and ROV methods was significant; the latter difference is likely due to whether or not bait is used to attract fish. Although the eDNA workflow implemented here requires improvements, the use of eDNA and imaging methods in combination provided better insight into the biodiversity of deep-sea fishes in the deep-sea around a seamount, where our knowledge of the fish fauna has been extremely limited. Our recovery of eDNA from seawater and sponge samples around the seamount demonstrates the potential of these methods for facilitating environmental baseline surveys and impact assessments of mining activities to obtain results not previously possible with the use of visual methods only.

## Introduction

Evaluating deep-sea biodiversity is essential to provide baseline data for assessing the environmental impacts of potential deep-sea mining activities^1,2^. Cobalt-rich ferromanganese crusts are widely distributed deep-sea minerals on seamounts, and mining of this resource has been explored^3,4^. Seamounts also support important deep-sea communities and may promote productivity that is critical for pelagic tuna and mackerel fisheries, as well as smaller-scale line fisheries in offshore areas^4–6^. However, the biodiversity of deep-sea communities on specific seamounts is still poorly understood because access to the deep sea for research is logistically difficult because of high costs and limited sampling opportunities^7^. Thus, further technological development for assessing biodiversity and environmental conditions around seamounts is needed.

Visual imaging techniques used for deep-sea biodiversity evaluation are non-invasive because they can be applied without collecting or significantly disturbing deep-sea organisms (versus, for example, bottom trawling)^8^, although light and noise may still affect the behaviour of deep-sea organisms to some extent^9,10^. Visual imaging by remotely operated vehicle (ROV) or autonomous underwater vehicle (AUV) is appropriate for surveying sessile organisms such as corals and sponges, which have been suggested to be especially vulnerable to deep-sea mining activities^11–13^. Mobile techniques, including the use of ROVs and AUVs, also record highly motile organisms such as fish, which are observed “naturally” along the survey lines, and the results can be used for density calculations. Species identification of fishes is, however, sometimes based on relatively small morphological features, such as the shape of a particular fin. Even when equipped with fairly high-resolution cameras, such mobile observation systems sometimes have difficulty capturing the small morphological features necessary for taxonomic identification of highly motile organisms. Camera landers such as the Edokko Mark I^14^ are limited by their static nature. Because they require organisms to come to the lander either by chance or by being attracted by bait, it is possible but more challenging to estimate densities from such data^15^. However, the advantages of a baited camera lander should not be overlooked. Fish that normally move rapidly may be attracted to the bait and thus stay in front of the camera, making detailed observations that allow higher taxonomic resolution possible. In addition, fish that are averse to the sound and light of ROVs and AUVs may be attracted to the bait of a camera lander. Furthermore, being able to record the feeding behaviour of fish and their attack behaviour directed against other organisms over food is an important advantage of camera lander imaging.

Environmental DNA (eDNA) analyses using modern high-throughput sequencers have enormously improved methods for biodiversity evaluation and solutions to environmental issues in both marine and freshwater environments^16–19^, and they are also being used in deep-sea ecosystems^20–23^. Environmental DNA analysis entails extracting DNA from environmental samples, amplifying and sequencing specific DNA regions, depending on the primers used, and then identifying the sequences by matching them against reference databases. For fish in particular, dedicated primers and databases are available, and eDNA can be used to identify a relatively large number of fish species^24,25^. The eDNA method is non-invasive and can be used to identify organisms in the vicinity of the sampler, sometimes at the species level, simply by sampling the water. Imaging methods using ROVs, AUVs, and camera landers are also non-invasive, but they may sometimes provide insufficient high-resolution taxonomic information for identification of fish and other organisms. The eDNA method, in contrast, is not only non-invasive but also has the potential to overcome the shortcomings of these imaging methods.

The number sequence reads required for eDNA analyses has been found to be correlated with biomass^26^, therefore, because biomass is low in the deep sea, filtering large volumes of seawater (more than a few liters) can improve the detection of deep-sea fish by DNA sequencing analyses^20^. On research vessels, however, eDNA analysis of deep-sea water is often difficult because of the limited amount of seawater available, which may be as little as 1–2 L per site; this limitation relates to the number and size of water samplers that can be mounted on the ROVs and the number of sampling sites per dive, as well as the fact that most of the sampled seawater is used for routine chemical oceanographic analyses. As a result, recent eDNA analyses have focused on sponges, which are natural eDNA samplers^27,28^ because they filter large volumes of seawater daily (more than several thousand liters of seawater per day)^27,29^. Thus, tissue from deep-sea sponges, which are widespread sessile organisms that create habitat complexity and therefore support biodiversity on seamounts^30,31^, has recently been used for the analysis of eDNA circulating around deep-sea areas, including seamounts^32,33^.

**Fig. 1:**
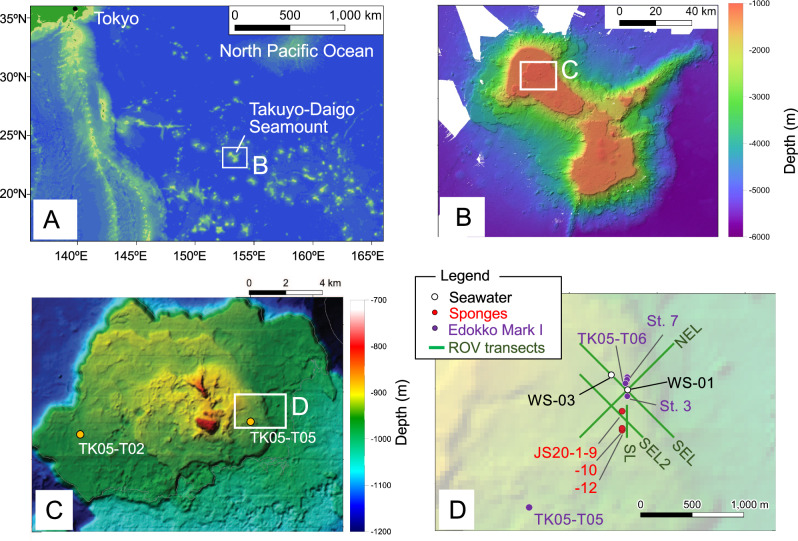
Study site. **A**, **B** Location of Takuyo-Daigo Seamount in the northwestern Pacific. **C** Edokko Mark I deployment stations. **D** ROV video transects and water sampling stations. The Edokko Mark I was deployed at stations TK05-T02 and TK05-T05 during the JK19 cruise in 2019, and the other surveys were conducted in 2020.

Here, through the collection of seawater and sponge samples from Takuyo-Daigo Seamount in the northwestern Pacific (Fig. 1, Tables 1–2, Supplementary Figs. 1–2), we use eDNA analyses to assess the composition of deep-sea fish communities by using primers targeting a hypervariable region of the 12 S rRNA gene (MiFish), which allows fish species identification^24^. We compare the eDNA results with those of methods using imagery obtained at the same time as the water and sponge sampling by an Edokko Mark I camera lander and an ROV (Tables 3 and 4). Although Takuyo-Daigo Seamount has been highlighted for its potential for future deep-sea mining^34^, information on the biodiversity of this seamount has been lacking. Thus, a baseline survey is urgently needed.

**Table 1:** Seawater eDNA samples

Sample ID	Collection date and time	Station	Altitude above seafloor (m)	Filtered volume (L)
JS20-1-W3	2020/6/16 14:58	WS-01	1.6	1.0
JS20-1-W4	2020/6/17 8:53	WS-01	3.0	1.0
JS20-1-W5	2020/6/19 8:44	WS-01	1.5	1.0
JS20-1-W7	2020/6/20 12:58	WS-01	3.0	2.35
HK20-3-WS1	2020/7/7 –	WS-01	1.5	0.95
HK20-3-WS2	2020/7/9 15:02	WS-01	5.1	0.95
HK20-3-WS7	2020/7/15 10:16	WS-01	4.6	1.0
JS20-2-W1F	2020/8/8 14:16	WS-01	3.0	1.0
JS20-2-W1	2020/8/8 14:16	WS-01	3.0	1.0
JS20-2-W3	2020/8/9 9:31	WS-01	3.0	3.0
JS20-2-W4	2020/8/15 11:43	WS-01	1.6	1.0
JS20-2-W6	2020/8/17 9:27	WS-03	3.3	1.0

Seawater samples were collected with 5-L Niskin bottles mounted on an ROV and filtered through 0.22 µm Sterivex filters. Dates and times are according to Japan Standard Time (UTC + 09:00). The water depth at stations WS-01 and WS-03 is ~934 m.

**Table 2:** Sponge samples, including accession numbers, and wet weight of tissue used for eDNA analysis

Sample ID	Taxonomic name	Accession No.	Wet weight (mg)
JS20-1-9	*Farrea* sp.	LC628640	64.7
JS20-1-10	*Pheronema* sp.	LC628642	232
JS20-1-12	*Geodia* sp.	LC628641	395

The water depth at the collection sites is ~936 m.

**Table 3:** Edokko Mark I free-fall camera lander deployments

Station	Depth (m)	Video recording period at seafloor			Period (d)	Video recording time (min)	Remarks
TK05-T02	949	2019/10/12 14:41	–	2019/10/16 7:01	4	403	Baited
TK05-T05	930	2019/10/13 9:52	–	2019/10/17 5:31	4	426	Baited
TK05-St.3	938	2020/6/15 11:59	–	2020/7/31 0:59	46	786	Not baited
TK05-St.7	937	2020/6/15 11:59	–	2020/7/31 8:00	46	786	Not baited
TK05-T06	938	2020/8/16 9:59	–	2020/8/20 7:59	4	95	Baited

Dates are according to Japan Standard Time (UTC + 09:00).

**Table 4:** ROV transect lines on the summit of Takuyo-Daigo Seamount

Transect	Date	Start and end time			Observed period (min)	Observed distance (m)
JS20-1-NSL3S	2020/6/19	9:07	–	10:27	80	155
JS20-1-SEL	2020/6/20	9:49	–	11:45	116	1,273
JS20-2-NSL3S	2020/8/19	8:57	–	9:16	19	155
JS20-2-NWL2	2020/8/19	10:17	–	14:43	266	849
JS20-2-NEL	2020/8/20	10:06	–	15:24	318	1,273

Dates are according to Japan Standard Time (UTC + 09:00).

## Results

### Imaging fish diversity

**Fig. 2:**
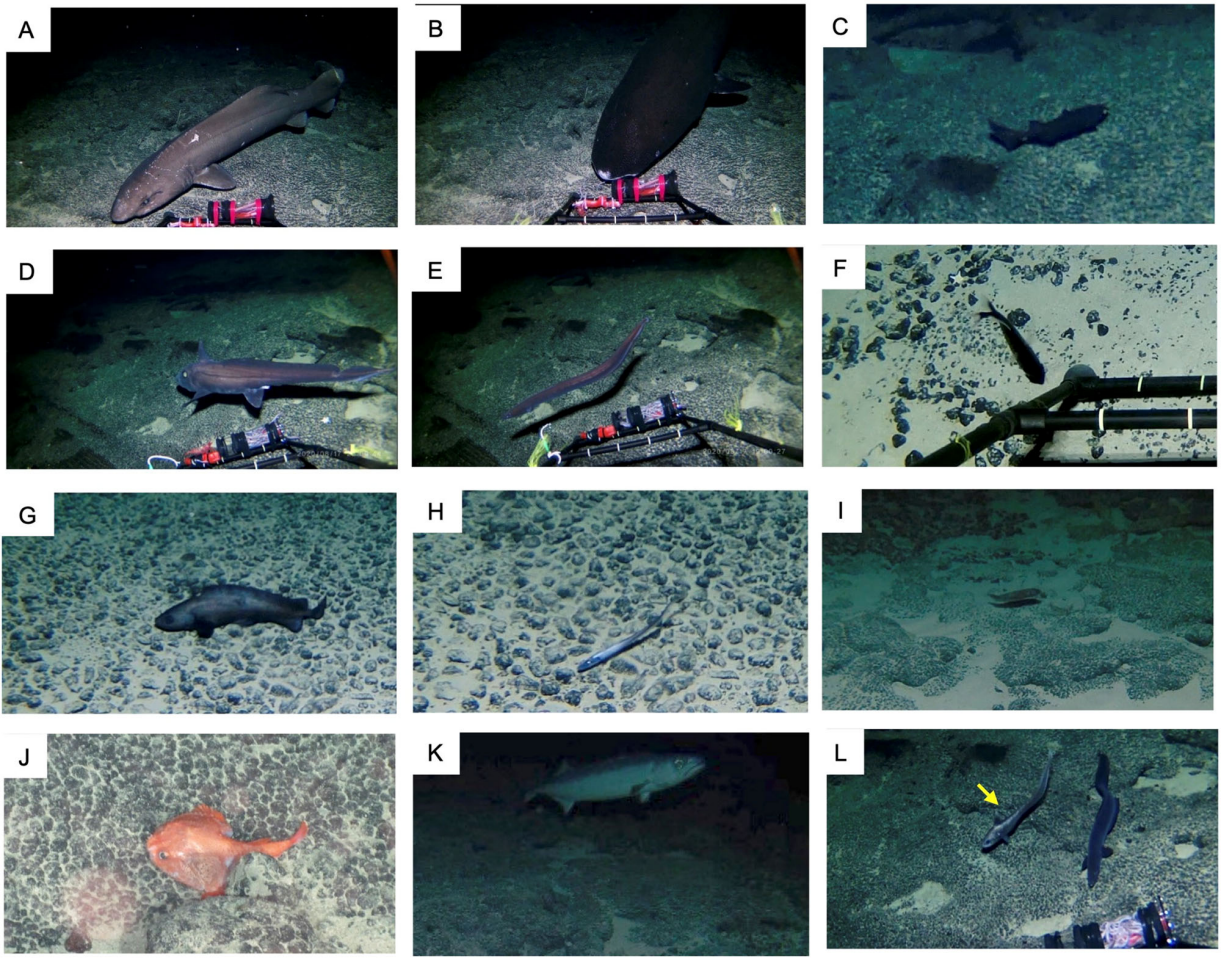
Deep-sea fishes observed by ROV (photo J) and the Edokko Mark I (all others) on the plateau of the Takuyo-Daigo Seamount. **A** Pseudotriakidae (*Pseudotriakis microdon*), **B** Somniosidae (*Somniosus pacificus*), **C** Etmopteridae, **D** Chimaeridae, **E** Synaphobranchidae, **F** Myctophidae, **G** Moridae, **H** Halosauridae, **I** Ophidiidae, **J** Chaunacidae, **K** Gempylidae (*Ruvettus pretiosus*), **L** Macrouridae. The width of the ladder is 60 cm.

**Fig. 3:**
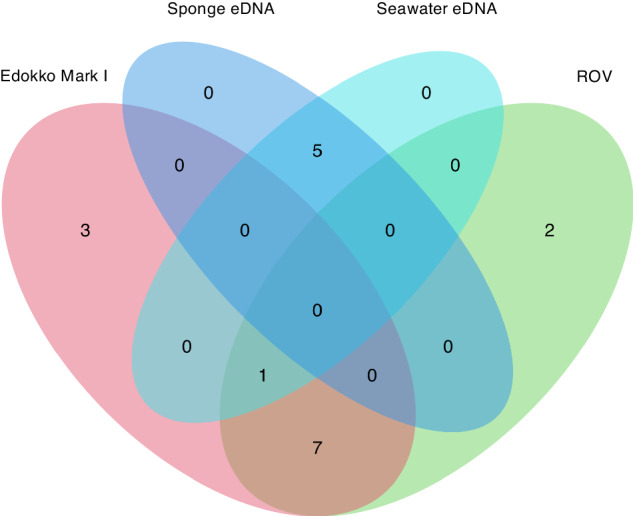
Venn diagram at family level of deep-sea fishes detected by four methods: eDNA analysis of seawater and sponge tissue, imagery analysis of the Edokko Mark I camera lander and ROV transect.

Observations were successfully conducted on Takuyo-Daigo Seamount in the northwestern Pacific (Fig. 1, Tables 3–4) using an ROV and an Edokko Mark I free-fall deep-sea camera lander. In the imaging analysis, we identified deep-sea fishes to species as finely as possible (Fig. 2, Table 5), and compared the results with the eDNA analysis results at the family level (Table 6 and Supplementary Table 1). Using the Edokko Mark I imaging method, we detected a total of 11 families of deep-sea fishes, including Macrouridae, whereas using the ROV transect method we detected 10 families, including Halosauridae and Synaphobranchidae (Table 6, Supplementary Table 1). Eight families were identified by both methods (Fig. 3) .

**Table 5:** Faunal list of deep-sea fishes detected by four methods: analysis of imagery obtained with the Edokko Mark I camera lander or ROV transect, and analysis of eDNA in seawater and sponge tissue

Order	Family	Species	Standard Japanese Name	Eddokko Mark I	ROV	Sponge eDNA	Seawater eDNA
Carcharhiniformes	Pseudotriakidae	*Pseudotriakis microdon* de Brito Capello 1868	Chihirozame	+			
Squaliformes	Etmopteridae	Etmopteridae gen. sp.	Karasuzame-ka	+	+		
	Somniosidae	*Somniosus* (*Somniosus*) *pacificus* Bigelow & Schroeder 1944	Ondenzame	+			
		Squaliformes gen. sp. 1	Tsunozame-moku	+			
		Squaliformes gen. sp. 2	Tsunozame-moku	+			
Chimaeriformes	Chimaeridae	*Hydrolagus* sp.	Aka-ginzame-zoku	+			
Notacanthiformes	Halosauridae	Halosauridae sp.	Tokagegisu-ka	+	+		
		Notacanformes sp.	Sokogisu-moku	+	+		
Anguilliformes	Synaphobranchidae	*Simenchelys parastica*	Kongou-anago	+	+		
		*Synaphobranchus* sp.	Hora-anago-zoku	+			
		Synaphobranchidae gen. sp.	Hora-anago-ka		+		
		Anguilliformes sp.	Unagi-moku		+		
Stomiiformes	Gonostomatidae	*Sigmops elongatus* (Günther 1878)	Oo-yokoeso			+	
		*Sigmops* sp.	Yokoeso-zoku				+
Myctophiformes	Myctophidae	Myctophidae gen. sp.	Hadakaiwashi-ka	+	+		
Gadiformes	Macrouridae	Macrouridae gen. sp.	Sokodara-ka	+	+		
	Bathygadidae	*Bathygadus antrodes* (Jordan & Starks 1904)	Ana-dara			+	+
	Moridae	*Antimora microlepis* Bean 1890	Canada-dara	+			
		*Physiculus* sp.	Chigodara-zoku		+		
Ophidiiformes	Ophidiidae	Ophidiidae gen. sp.	Ashiro-ka	+	+		
Lophiiformes	Chaunacidae	Chaunacidae gen. sp.	Fusaankou-ka		+		
Beryciformes	Melamphaidae	*Poromitra* sp.	Kabutouo-zoku				+
		*Scopeloberyx robustus* (Günther 1887)	Minami-tatekabutouo			+	+
		*Scopelogadus mizolepis mizolepis* (Günther 1878)	Yoroi-ginme			+	+
Cetomimiformes	Barbourisiidae	*Barbourisia rufa* Parr 1945	Akakujirauo-damashi			+	+
Acropomatiformes	Howellidae	*Howella zina* Fedoryako 1976	Togekushi-sumikuiuo			+	+
Perciformes	Scorpaenidae	Scorpaenidae gen. sp.	Fusakasago-ka		+		
	Gempylidae	*Epinnula rex* Ho, Motomura, Hata & Chiang 2017	Ao-sumiyaki				+
		*Ruvettus pretiosus*	Baramutsu	+	+		
		*Promethichthys prometheus* (Cuvier 1832)	Kuroshibikamasu				+

**Table 6:** Occurrence patterns of deep-sea fishes at the family level detected by the four methods: analysis of imagery obtained with the Edokko Mark I camera lander or ROV transect, and analysis of eDNA in seawater and sponge tissue

Class		Methods			
Family		Edokko Mark I	ROV	Sponge eDNA	Seawater eDNA
Chondrichthyes					
	Pseudotriakidae	+			
	Etmopteridae	+	+		
	Somniosidae	+			
	Chimaeridae	+			
Osteichthyes					
	Halosauridae	+	+		
	Synaphobranchidae	+	+		
	Gonostomatidae			+	+
	Myctophidae	+	+		
	Macrouridae	+	+		
	Bathygadidae			+	+
	Moridae	+	+		
	Ophidiidae	+	+		
	Chaunacidae		+		
	Melamphaidae			+	+
	Barbourisiidae			+	+
	Howellidae			+	+
	Scorpaenidae		+		
	Gempylidae	+	+		+
	Number of families	11	10	5	6

### eDNA fish diversity

We analyzed eDNA of seawater and sponge samples collected at the same time as the image-based fish diversity survey was carried out (Fig. 1, Tables 1–2). We sampled sponges belonging to three taxa, identified morphologically and by COI sequencing to be from *Farrea* sp., *Pheronema* sp. and *Geodia* sp. (Table 2, Supplementary Tables 2–3). The seawater samples generally exceeded 20,000 reads per sample in eDNA analysis, but a single sample had lower than expected number of reads (5000 for JS20-2-W1; Supplementary Tables 2–3). Fish eDNA was detected in all sponge and seawater samples; both deep-sea and shallow-water fishes (e.g., subtropical Siganidae species) were detected. Our eDNA analysis easily detected four families (up to 16 species) in the sponge samples (Supplementary Tables 1–2), whereas in the seawater samples, at most one taxon (up to 3 species) was identified (Supplementary Tables 1, 3). Of all taxa detected in the sponge and seawater samples, shallow-water species accounted for 69% and 18%, respectively. In other words, deep-sea fishes accounted for 82% of the fish taxa detected in the seawater samples. Although the seawater and sponge samples were collected at nearby sites, the eDNA analysis results were contrasting: In the seawater samples, deep-sea fish were more selectively detected, whereas in the sponge samples, the number of fish species that could be detected was higher. These biases should be kept in mind and interpretation should be conservative. In the downstream analysis, we focused on only deep-sea fishes. We assessed their phylogenetic relationships with reference fish sequences (Supplementary Figs. 4–8), which supported the MiFish pipeline results of species identification. The eDNA analyses detected seven deep-sea fish families in the seawater samples and five in sponge samples (Table 6, Supplementary Table 1). In the eDNA analysis results, diversity tended to be saturated in all samples (Supplementary Fig. 9), but this is due to the small number of species originally detected.

### Method comparison

**Fig. 4:**
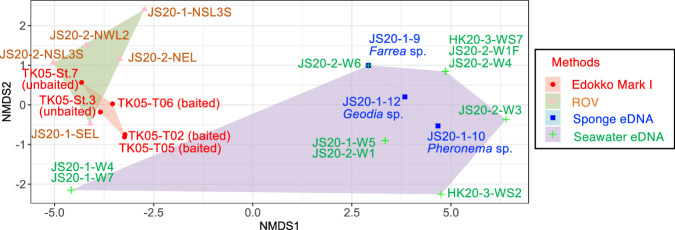
Nonmetric multidimensional scaling (nMDS) plot of deep-sea fishes using Jaccard similarities. Presence/absence data of the methods were used. The colours of the convex hull denote methods: eDNA analysis of seawater (purple) and sponge tissue (light blue), imagery analysis of the Edokko Mark I camera lander (pink) and ROV transect (light green). Two-dimensional stress value = 0.0151.

The number of deep-sea fish families detected by each sample type and approach are shown in Table 6. A Venn diagram at the family level of deep-sea fishes shows that a total of 18 families were detected, but no family was detected by all four methods (Fig. 3). Nonmetric multidimensional scaling (nMDS) revealed three distinct clusters (Fig. 4; Stress value: 0.0151) that corresponded to the taxa detected by Edokko Mark I and ROV imaging and by seawater eDNA analysis, whereas the sponge eDNA cluster overlapped completely with the seawater eDNA cluster (Fig. 4). Permutational multivariate analysis of variance (PERMANOVA) detected significant differences in fish compositions between the imaging and eDNA analyses and between the Edokko Mark I and ROV imaging methods, but not between seawater and sponge eDNA methods (Table 7). Indicator species and SIMPER analyses identified characteristic species associated with each method (Supplementary Tables 4–5).

**Table 7:** PERMANOVA analysis results for the effects of methods on community structure

Source	df	SS	Pseudo-F	P-value
All eDNA vs imaging	1	1.959	5.4143	0.0001
Edokko Mark I vs ROV	1	0.757	3.2076	0.0256
Seawater eDNA vs sponge eDNA	1	0.353	0.8439	0.5697

Presence/absence data of the methods were used. *df* degrees of freedom, *SS* sum of squares, *Pseudo-F* pseudo-F-statistic, *P* probability.

## Discussion

Through imaging by the Edokko Mark I and ROV, we were able to detect deep-sea fishes inhabiting the Takuyo-Daigo Seamount, most of which were common demersal deep-sea fish^5,6,35^. The fish fauna found on this seamount includes many families commonly found in deep-sea areas (e.g. Synaphobranchidae, Myctophidae) and is similar to the fauna of other seamounts in the northwestern Pacific located at a similar latitude (Ritto and Nikko Seamounts on the central and western Mariana ridges, Shoho and Shotoku Seamounts on Nishi-Shichito Ridge)^36^. The deep-sea fish groups that appeared in three of the four seamounts studied by Koeda et al.^36^ can be considered the general groups to be found in this region, which could be expected to be detected at Takuyo-Daigo Seamount. In fact, most of them were detected in our imaging survey. Of the 16 families recorded in the present study, eight families, or half, matched the ichthyofauna list from the Ritto Seamount with detection by the same way, representing 28.6% of the 28 families recorded from the Ritto Seamount in Koeda et al.^36^. This result suggests that our imaging survey methods using the Edokko Mark I camera lander sand ROV transects were generally appropriate.

Although the major groups of deep-sea fish were observed, three specific families, Squalidae, Chlorophthalmidae, and Setarchidae, were not be observed on Takuyo-Daigo Seamount. These families occur commonly on the Nisi-Shichito Ridge and the central and western Mariana ridges^36^. Although both this study and that of Koeda et al. employed ROVs and camera landers in combination, the total observation time of our ROV survey (~13.3 h, Table 4) was shorter than that of Koeda et al. (55.2 h)^36^. If a longer observation time would allow more species to be detected, then we may have missed the three families because our observation time was too short. However, the search method used may also be a factor. Whereas Koeda et al.^36^ used an opportunistic/exploratory ROV survey method^36^, this study employed a transect method between fixed stations. The three families not detected in this study are benthic and do not active swimmers. Therefore, we consider that they may be less likely to be observed by the ROV transect survey method.

Interestingly, the fish compositions clearly differed between the imaging and eDNA methods. Among previous studies that have conducted simultaneous eDNA and visual surveys^32,37–39^, some have reported trends similar to that we found with little overlap between eDNA and visual survey results^32,39^.We attribute this lack of overlap to variations in the power of detection of some fish groups between visual and eDNA methods (e.g., variations in primer compatibility and detectable fish size). Furthermore, according to Miya et al.^24^, the MiFish primers cannot amplify eDNA from a number of elasmobranchs (sharks and rays), which may explain why our eDNA analyses did not detect four families belonging to class Chondrichthyes that were captured by the Edokko Mark I camera landers (Table 6).

Similar mismatches between eDNA and imaging method results have been reported previously^40,41^. Differences in the amount of DNA released by different organisms have been suggested as one reason for some organisms not being easily detectable by eDNA methods^38^; in this case, however, because fish were the target species, such difference may not be relevant. Five fish families of deep-sea fish, Gonostomatidae, Bathygadidae, Melamphaidae, Barbourisiidae, and Howellidae, that were detected by our eDNA analyses were not detected by our imaging methods. Interestingly, these five families were also not detected by the imaging method using by Koeda et al.^36^. These families are widely distributed in the deep sea, including in the northern Pacific, so it is difficult to believe that they are not distributed in the study areas of Koeda et al.^36^. Alternatively, these fishes, which are <10 cm long^42^, may be too small to capture in images. Fish with small body size that swim in schools at altitudes greater than 1 m above the seafloor are reported to be sensitive to ROVs^10,43^. These lines of evidence support our contention that different taxa can be detected by an eDNA approach than can be detected by imaging methods. The simultaneous application of imaging and eDNA methods is therefore expected to provide a better picture of the fish fauna at the time of the survey on Takuyo-Daigo Seamount.

Significant differences in fish composition between the Edokko Mark I camera lander and ROV transect methods were found (Table 6). Although the Edokko Mark I captured several families in class Chondrichthyes, the ROV observations showed few fishes in this group (Supplementary Table 1). As a results, the convex hulls corresponding to the two imaging methods seen in the nMDS plot do not completely overlap (Fig. 4). Differences in the sensitivities of fishes to moving vehicles or lighting have been reported^44–46^, but most families observed in the present study have been reported to be non-responsive to ROVs^47^. There are no reports on the avoidance behaviour of Pseudotriakidae, nor on Somnioidea, and these families were not observed by the ROV in this study. It is therefore unclear whether the differences observed between the ROV and Edokko Mark I in this study reflect avoidance behaviour of fishes. Also, the ease of attraction to bait in certain fish groups may be related to the distinction between imagery methods. Pseudotriakidae, and Somnioidea sharks have been observed only at bait landers, and they may have been attracted to the baits (Supplementary Table 1); thus, they may not be easily observed at unbaited landers or ROVs. Chaunacidae were detected only by ROV (Table 6 and Supplementary Table 2); it is likely that the Edokko Mark I could not detect this group, which tends to be philopatric^48^, and thus less mobile, within the narrow observation range. Both the Edokko Mark I and ROV methods enabled us to observe a wider range of fish groups, but future surveys at other seamounts are needed to confirm these observation patterns.

Our eDNA analysis results showed no difference in fish composition between the seawater and sponge samples (Table 7). The three sponge samples each belonged to a different family, but we obtained only one specimen of each. Therefore, it is difficult to make any quantitative observations about the relationship between the type of sponge and the fish species that could be detected. However, with reference to previous reports, we can discuss the possible influence of the type of sponge on the eDNA analysis results^27,32^. Morphological differences among sponge species such as of body wall thickness might have influenced the number of fish families detected: only one fish family was detected in specimen JS20-1-9 (*Farrea* sp.), which has the thinnest wall among the three sponge families (Table 6 and Supplementary Table 1). It has also been reported that the detection rate of fish eDNA differs significantly among sponge species, with the highest detection rates found for sponges with low microbial activity, such as sponges of class Hexactinellida^32^. Among the sponge samples used in this study, specimens JS20-1-9 (*Farrea* sp.) and JS20-1-10 (*Pheronema* sp.) belong to Hexactinellida, but JS20-1-9 (*Geodia* sp.) belongs to class Demospongiae. Indeed, the highest number of fish families among the three specimens was detected in specimen JS20-1-10 (*Pheronema* sp.) (Supplementary Table 1).

Surveys evaluating the biodiversity of deep-sea fishes and corals rely on promising eDNA solutions^20,49^, and eDNA analysis of sponge tissue offers a promising way to evaluate deep-sea biodiversity around seamounts. McClenaghan et al. reported that the power to detect deep-sea fishes by using eDNA depends on the volume of the seawater sample^20^. Although increasing the sample volume for eDNA analyses around seamounts is desirable, in practice, it is often difficult to collect even a few litres of deep-sea water, which is generally required the minimum volume required for eDNA analysis. Previous studies has proposed using eDNA methods with sponge samples in combination with other methods for biodiversity assessment^32,33^. The relationship seen in the seawater eDNA method, that the detection rate increases with the volume of seawater sample analyzed, appears to also hold for sponge samples. Because only a few hundred milligrams of sponge tissue from each specimen was used in this study (Table 2; other studies have used 0.25 g of sponge tissues^32^ or a maximum tissue weight of 500 mg^50^), it would be easy to increase the amount of tissue analyzed for eDNA. In the future, more tissues should be used for eDNA analysis and a larger number of sponge colonies should be tested. We expect sponge colonies to be located in favourable currents for resource availability. Therefore, future studies should also assess how the location preferences of sponges relate to spatial variability in fish detection results obtained by using sponge eDNA.

The use of eDNA and imaging methods in combination should provide a more comprehensive view of deep-sea fish biodiversity around seamounts than either type of method used singly^51^. Our trial study showed the potential of eDNA analyses using seawater and sponge samples to facilitate environmental baseline surveys and impact assessments around seamounts. Yet it must be stressed that not only larger water volumes and sponge tissue amounts but also a larger number of samples must be processed. Indeed, the limited number of samples used in this study notably reduced the robustness of our statistical analyses. More importantly, neither eDNA or imaging methods alone are sufficient for capturing a complete picture of fish diversity, because each method misses certain types of fish. Therefore, fish diversity surveys that do not use complementary methods must always be insufficient. This important finding has implications for future studies. Recently, Kopp et al. have underlined the importance of complementary survey methods by showing examples of cnidarians and bryozoans that are difficult to detect by the eDNA method but are easily captured by video observation^38^. Their result is intriguing, and we are in agreement that complementary survey methods are important, even though the difference in their results are at the level of the phylum and those in our results are at the level of fish family.

Our eDNA analysis detected not only deep-sea fishes but also shallow-water fishes of subtropical regions (e.g., Siganidae species), which may indicate that contamination occurred when the samples were pulled up through the surface seawater. Although we used the normal methods to collect seawater and biological samples, they were not adequate to prevent such contamination. Judging from the results of the eDNA analysis, we believe that the contamination of the seawater samples with the DNA of shallow-water fish species was minimal and that the most significant contamination was in the sponge samples. Therefore, a response measure that should be considered in the future is to collect sponge samples on the seafloor into a well-sealed collection container so that they do not come into contact with surface seawater while being raised to the surface. Environmental RNA analysis would also be useful for distinguishing contamination^52,53^. Given the low-temperature environment of deep-sea waters, eRNA methods should be considered to distinguish legacy eDNA (extracellular or non-living material^54^) from autochthonous, contemporary eDNA. In addition, improvements such as the use of a wider array of primers for detecting more fishes and higher resolution imaging should be pursued to understand the spatial variability of the results obtained with each method used in this study around seamounts and to identify taxa at lower taxonomic levels (genus or species level). Because the results of the eDNA and imaging methods did not overlap much, the newly developed method should also be implemented simultaneously with the imaging methods to provide a comprehensive picture of the diversity of fish communities around seamounts.

Although mining tests have been conducted at the Takuyo-Daigo Seamount and the effects on turbidity and fish have been investigated^55,56^, baseline information on the fish fauna at this seamount is still lacking. Knowledge of the fish fauna of the Marcus Wake seamount group and the Ogasawara Islands, which include the Takuyo-Daigo Seamount, is extremely poor. In addition, most biodiversity surveys in deep-sea areas have not been comprehensive because of technical limitations of the methods used and the lack of sufficient survey time and coverage. Given the lack of knowledge of the fish fauna at this seamount, the publication of the species composition detected in this study and its analysis based on the current results will contribute to the assessment of biodiversity in this region and to the consideration of appropriate conservation measures. The approach taken in this study will be helpful for conducting a comprehensive fish fauna baseline surveys before any future deep-sea mining activity is undertaken at deep-sea seamounts.

## Study sites and methods

### Takuyo-Daigo seamount

The seamount is a relatively large guyot (150 km wide), and its summit consists of two plateaus (Fig. 1). The shallowest water depth on the summit is 810 m, and the seamount stands about 4500 m above the surrounding abyssal plain (~5300 m water depth). Vertical profiles of temperature, salinity, dissolved oxygen (DO) concentration, and turbidity were observed at two stations at the base and on the flat top of the seamount (Supplementary Fig. 1). Our study site was located within the central part, in terms of depth, of a naturally occurring oxygen minimum zone (OMZ)^57,58^.

### Study site for Edokko Mark I

We performed environmental surveys of the Takuyo-Daigo Seamount (Fig. 1) from the research vessels *Kaiyo Maru No. 1* (cruise id: JK19), *Hakurei* (cruise id: HK20-3), and *Shinsei Maru* (cruise id: JS20-1, JS20-2) between autumn of 2019 and summer of 2020. Imaging surveys with the Edokko Mark I benthic lander (Okamoto Glass Co., Ltd., Kashiwa, Japan) were performed at five sites on the more northerly plateau (Fig. 1C, D; Table 3). The water depths at the five sites were very narrow, ranging from 937–949 m. We have three sets of Edokko Mark I lander, which we use alternately, all the same size and with the same model camera, and we consider the instrumental differences negligible. Detailed configurations and protocols of operations of the Edokko Mark I benthic lander are described by the Japan Agency for Marine-Earth Science and Technology^59^. Briefly, the main body of the lander is composed of three glass spheres, for the transponder, lighting, and cameras, respectively (Supplementary Fig. 2C). Video images were obtained by digital high-definition (HD) cameras installed in the camera sphere with LED lighting provided by the lighting sphere. The camera sphere was placed at a height of 1 m above the seafloor and captured images of the seafloor with two cameras with a 58° vertical angles of view and pointing downward at angles of about 30° and 60°. In this study, both cameras were used for fish identification. The benthic camera lander was baited with Pacific saury (*Cololabis saira*) attached to the lower ladder during three 4 day periods at stations TK05-T02, TK05-T05, and TK05-T06 (Table 3). Video shooting was scheduled at TK05-T02 and TK05-T05 continuously for ~5 h from deployment and then thereafter for 1 min every 1 h until retrieval. For the deployment at TK05-T06, video was recorded for 1 min every 1 h from deployment until retrieval. At TK05-St. 3 and TK05-St. 7, the benthic landers were deployed for ~40 days without bait. Video shooting was scheduled for 1 min every 4 h for the first 12 days and then for 1 min every 1 h thereafter until retrieval.

### Study site for ROV transect surveys

Video surveys were performed along four transects on the Takuyo-Daigo Seamount plateau (Fig. 1D; Table 4) by the working-class ROV “Hakuyo 3000” of Fukada Salvage & Marine Works Co., Ltd., during *Shinsei Maru* cruises JS20-1 and JS20-2 (Supplementary Fig. 2A)^55^. The speed of the ROV along the transects was ~0.5 kt. An HD camera (Insite Pacific Mini Zeus, Insite Pacific Inc., Solana Beach, CA, USA) mounted obliquely (downward angle: 20°) on the front of the ROV was used for video recording; the camera altitude was ~1.5 m above the seafloor. Parallel green laser lines with a width of 50 cm were used.

### Imaging analysis

All fish captured in the Edokko Mark I and ROV imagery were annotated with VLC Media Player and identified by reference to Fujikura et al.^60^ and other available information on fish classification^61,62^. The fish were identified to the lowest taxonomic level to which they could be assigned with confidence, if possible to the species level, but at least to the family level. In the presented analysis, for consistency among methods and to allow comparisons, only the family level identifications were used.

### Seawater sampling

The ROV “Hakuyo 3000” was used for water sampling during *Shinsei Maru* cruises JS20-1 and JS20-2, but another working-class ROV was used during the *Hakurei* cruise in July 2020. A total of 12 seawater samples were collected over two sites (WS-01, *n* = 11; WS-03, *n* = 1; Fig. 1D; Table 1) in 5 L-Niskin bottles attached to the ROV. Once the ROV was back on board, the Niskin sampler was removed and the seawater for eDNA processing was immediately aliquoted on deck in a well-ventilated area to reduce the potential for sample contamination (Supplementary Fig. 2F). All seawater samples (0.95–3.0 L) were filtered through 0.22-μm Sterivex filters (Supplementary Fig. 2G, MilliporeSigma, Burlington, MA, USA). During cruises JS20-1 and JS20-2, filtration was carried out using a Rocker Alligator 200 diaphragm liquid pump or EZ-Stream vacuum pump in an air-conditioned laboratory onboard. A custom-built filtration set-up was used. Normal filtration was completed within about 2 h of water sampling, although some samples took longer. Only one seawater sample was collected for eDNA analysis per site. Most samples were collected at the same location (WS-01) by repeated dives of the ROV. Filter cartridges were stored at –20 °C for the duration of the expedition and then transferred to an on-land laboratory before DNA extraction. Of the 12 seawater samples, four were treated in different ways. Water sample JS20-2-W1F was then frozen at –20 °C and then shipped frozen to the lab for subsequent processing, and three other seawater samples (HK20-3-WS1, HK20-3-WS2, and HK20-3-WS7) were refrigerated at 5 °C and shipped at that temperature to an onshore laboratory. Water filtration of the samples was conducted within 1 month after collection in a laboratory using a filtration set-up similar to that onboard the research vessel. The analytical results of these four samples that were handled differently from the standard method were also included in this study. Before each filtration session, all equipment was decontaminated with bleach and rinsed with deionized water. We also collected negative control samples for seawater eDNA analysis by filtering purified water (Invitrogen UltraPure DNase/RNase-Free Distilled Water, Fisher Scientific, Waltham, USA) in the same manner as the experimental samples two times during a cruise. Basic procedures such as preventing surface contamination using foaming bleach were conducted following Minamoto et al.^63^.

### Sponge sampling

Three sponge samples were collected with the ROV manipulator (Table 2, Fig. 1D and Supplementary Fig. 2H–K) and placed in customized plastic boxes near the deep-sea bottom. The collected sponges were fixed in 99.5% ethanol onboard. The sponge taxa were determined by morphological and genetic analyses as described below. The samples were identified taxonomically by the morphological characters of their siliceous spicules^64^ as well as by partial mitochondrial cytochrome c oxidase subunit I (COI) gene sequences obtained by polymerase chain reaction (PCR) with universal primers^65^ and the Sanger dideoxy sequencing method. For the COI gene analysis, small tissue pieces were separated from the sponge samples and washed several times with ethanol. DNA extraction was performed using a DNeasy Blood & Tissue Kit (QIAGEN) following the protocol provided with the kit. We used the same primers as Geller et al.^65^ (gLCO1490: TITCIACIAAYCAYAARGAYATTGG; jgHCO2198: TAIACYTCIGGRTGICCRAARAAYCA) for the COI analysis. The PCR analysis was performed in a 20-µL volume containing 2 µL 10× PCR buffer, 1.6 µL dNTPs, 1.2 µL of each primer (10 µM), 0.2 µL TaKaRa Ex Taq^TM^ Hot Start Version (Takara Bio Inc., Kusatsu, Japan), 12.8 µL purified water (Invitrogen UltraPure DNase/RNase-Free Distilled Water), and 1 µL DNA template. The PCR cycles were 1 min at 94 °C and then five cycles of 94 °C for 40 s, 45 °C for 40 s, and 72 °C for 1 min, followed by 35 cycles of 94 °C for 40 s, 51 °C for 40 s, and 72 °C for 1 min, and a final step of 72 °C for 7 min, following Radulovici et al.^66^. The PCR amplification products were subjected to electrophoresis to confirm that the DNA of the target base length had been amplified. The PCR amplification products were purified using ExoSAP-IT™ PCR Product Cleanup Reagent (Applied Biosystems) and then sequenced using the Sanger dideoxy sequencing method. The determined sequences were BLAST^67^-searched to estimate closely related species, and molecular phylogenetic analysis was performed to estimate the taxonomic groups to which the sponge species should be attributed.

### DNA analysis

We extracted DNA from the Sterivex filters by using a Qiagen DNeasy Blood and Tissue Kit according to the manufacturer’s protocol with some modifications^65^. Sponge DNA was extracted with a Qiagen DNeasy PowerSoil Kit from 3–5 tissue samples per individual for replicates (Supplementary Table 1). We used MiFish primers, which amplify a <200 bp region of the 12 S rRNA gene^24^, including an overhang adaptor sequence to amplify the target region of the eDNA of fishes for first-round PCR (1st PCR) in a total volume of 20 µL using Ex Taq^TM^ Hot Start polymerase (Takara Bio Inc., Kusatsu, Japan). The same concentration (10 µM) of each forward (Mifish-U-F and Mifish-E-F) or reverse primer (Mifish-U-R and Mifish-E-R) was mixed in equal amounts and used as the MiFish-U/E-F or MiFish-U/E-R primer for PCR following Miya et al.^24^. We used triplicate PCR amplicons for amplicon sequencing. The PCR was performed in a 20 µL volume containing 2 µL 10 × PCR buffer, 1.6 µL dNTPs, 1.0 µL of each primer (10 µM), 0.2 µL TaKaRa Ex Taq^TM^ Hot Start Version, 13.2 µL purified water (Invitrogen UltraPure DNase/RNase-Free Distilled Water), and 1 μL DNA template. The 1st PCR procedure consisted of initial denaturation for 2 min at 94 °C; 35 cycles of 20 s at 94 °C, 15 s at 65 °C, and 20 s at 72 °C; and a final 7 min at 72 °C. We obtained 1st PCR products from seawater and sponge DNA samples but not from negative controls. The PCR products were checked for base length of the target size by 2% agarose gel electrophoresis. SYBR^TM^ Gold Nucleic Acid Gel Stain (Invitrogen) was used for staining. Specifically, a 2% agarose gel (Agarose S, NIPPON GENE Co., Ltd., Tokyo, Japan) was set in a submarine electrophoresis system (Mupid-2 plus, Mupid) containing 1 × TAE buffer (50 × TAE, NIPPON GENE Co., Ltd., diluted at time of use) and electrophoresed at 100 V for 25 min. The electrophoresis was carried out at 100 V for 25 min. The electrophoresed agarose gels were stained with a 10,000 fold dilution of SYBR^TM^ Gold Nucleic Acid Gel Stain and checked with a UV transilluminator (FAS-V, NIPPON Genetics Co., Ltd., Tolyo, Japan) for DNA amplification products of the expected base length as bands. The bands were checked for the presence of DNA amplification products of the expected base length. Amplicons were purified on AMPure XP beads (Beckman Coulter, Brea, CA, USA). The sequencing adaptors with Illumina MiSeq sample index sequences were added to purified PCR amplicons in a second-round PCR (2nd PCR). The PCR was performed in a 20 µL volume containing 2 µL 10 × PCR buffer, 1.6 µL dNTPs, 1.6 µL of each primer (5 µM), 0.1 µL TaKaRa Ex Taq^TM^ Hot Start Version, 12.1 µL purified water (Invitrogen UltraPure DNase/RNase-Free Distilled Water), and 1 μL DNA template. The 2nd PCR procedure consisted of an initial 1 min at 96 °C; 15 cycles of 30 s at 96 °C, 45 s at 65 °C, and 1 min at 72 °C; and a final 7 min at 72 °C. After confirmation of amplification of the index PCR samples, the amplified product was purified with AMPure XP beads and pooled to an equimolar concentration (4 nM). Fragment size was confirmed by electrophoresis. Then, a library was prepared for 150 bp paired-end sequencing using MiSeq in a laboratory of our research group at AIST.

### Genetic identification

To identify fish groups from the paired-end sequences, we used the MiFish pipeline^25,68^ with default settings. Briefly, the low-quality tails were trimmed from each read (Phred score < 10), the tail-trimmed paired-end reads were assembled, and the primer sequences were then removed; sequences with 99% identity were considered to be identical, and BLASTN^67^ searches were conducted for annotation. To confirm the taxonomic positions of the fishes and sponges, we constructed molecular trees (Supplementary Figs. 3–8) from the obtained sequences (fish trees were obtained after processing by MiFish pipeline and sponge trees were obtained by Sanger sequencing) with taxonomic neighbours from the NCBI database by using the Kimura 2-parameter model and the neighbour-joining method^69^ in MEGA X software^70^. The confidence of each branch was evaluated by bootstrap test (1000 replications)^71^. All FASTQ files obtained in this study have been deposited in the DDBJ database (accession nos. DRA011832 and DRA011833). Community similarity analysis was performed by nonmetric multidimensional scaling (nMDS) using presence/absence data of the methods based on the Jaccard dissimilarity index. To evaluate the significance of differences in fish compositions between methods, we performed a permutational multivariate analysis of variance (PERMANOVA) with a Jaccard dissimilarity index and 9,999 permutations. Presence/absence data of the methods were used. Statistical analyses were performed in R v. 4.3.2 software^72^ with the “vegan” package version 2.6.4^73^. We performed indicator species analysis by calculating indicator values to detect characteristic family associated with each method using the indval function in the “labdsv” package version 2.1.0^74^. In addition, we conducted SIMPER analysis^75^ with the vegan package. For eDNA data, we also calculated alpha species diversities of deep-sea fishes based on Hill numbers using the “iNEXT” package version 3.0.0^76^ in R.

### supplementary information

Supplementary Data 1

Supplementary Tables

Supplementary Figures

## Data Availability

Sequence data used in this study are accessible under DDBJ database (accession nos. DRA011832, DRA011833, LC628640-LC628642).

## Code availability

Correspondence and requests for codes should be addressed to Akira Iguchi.
