# Supplementary material for: Utilizing environmental DNA and imaging to study the deep-sea fish community of Takuyo-Daigo Seamount
Source: NPJ Biodivers. 2024 May 31;3:14. doi: 10.1038/s44185-024-00042-w (PMC11331990; doi:10.1038/s44185-024-00042-w)
Supplement: Supplementary file 1 — Supplementary Data 1 [file 44185_2024_42_MOESM1_ESM.pdf]

```
>JS20-2W1F_Seawater
CACCGCGGTTATACGAGCGACCCAAGCTGATAGTCATCGGCGTAAAGAGTGGTTAAGGGA
TAACATAAACTAAAGCCGAACGCCCTCAAGGCTGTTATACGCATCCGAGAGTACGAAGCT
CTAATACGAAAATGGCTTTAAACCCCACCTGATCCCACGAAAGCTACGCAA
>HK20-3WS7_SeaWater
CACCGCGGTTATACGAGCGACCCAAGCCGATAGCCCCGGCGTAAAGAGTGGTTAGGGGTC
CCTAAACTAAAGCCGAACGCTCTCAAAGCTGTTATACGCTTTCGAGGGTACGAAGCCCCA
CCACGAAAGTGACTTTAAATCTCCCTGAACCCACGAAAGCTAAGCAA
>JS20-2W4-2_Seawater
CACCGCGGTTATACGAGCAGCCCAAGCTGATAAATATCGGCGTAAAGAGTGGTTAAGGAG
TAAAACAAAATAAAGCCGAACGCCCTCAAGGCTGTTATACGCATCCGAAGGTACGAAGCA
CCCCTACGAAAGTAACTTTAAACCCCGCCTGACCCCACGAAAGCTACGAAA
>JS20-1_10-1-MiFish_Sponge
CACCGCGGTTATACGAGCAGCCCAAGCTGATAAATATCGGCGTAAAGAGTGGTTAAGGAG
TAAAACAAAATAAAGCCGAACGCCCTCAAGGCTGTTATACGCATCCGAAGGTACGAAGCA
CCCCTACGAGAGTAACTTTAAACCCCGCCTGACCCCACGAAAGCTACGAAA
>JS20-1_12-1-MiFish_Sponge
CACCGCGGTTATACGAGCGGCCCAAGCCGATAGCCCCGGCGTAAAGAGTGGTTAGGGGTC
CCTAAACTAAAGCCGAACGCTCTCAAAGCTGTTATACGCTTTCGAGGGTACGAAGCCCCA
CCACGAAAGTGACTTTAAATCTCCCTGAACCCACGAAAGCTAAGCAA
>JS20-1W5_Seawater
CACCGCGGTTATACGAGGGACCCAAATTGATAGCTACCGGCGTAAAGCATGATTGGGGGT
ACAACCAACTAAAGCCCAACCCACCCCCGCTGTCATACGCCTCCGGAAAAATGAAAACCC
ACCACGAAAGTAGCTTTAACCACACCTGAACTCATGACAACTAAGAAA
>JS20-2W1_Seawater
CACCGCGGTTATACGAGGGACCCAAATTGATAGCTACCGGCGTAAAGCATGATTGGGGGT
ACAACCAACTAAAGCCCAACCCACCCCCGCTGTCATACGCCTCCGGAAAAATGAAAACCC
ACCACGAAAGTAGCTTTAACCACACCTGAACTCATGACAACTAAGAAA
>JS20-1_10-5-MiFish_Sponge
CACCGCGGTTATACGAGGGACCCAAATTGATAGCTACCGGCGTAAAGCATGATTGGGGGT
ACAACCAACTAAAGCCCAACCCACCCCCGCTGTCATACGCCTCCGGAAAAATGAAAACCC
ACCACGAAAGTAGCTTTAACCACACCTGAACTCATGACAACTAAGAAA
>JS20-1_12-3-MiFish_Sponge
CACCGCGGTTATACGAGGGACCCAAATTGATAGCTACCGGCGTAAAGCATGATTGGGGGT
ACAACCAACTAAAGCCCAACCCACCCCCGCTGTCATACGCCTCCGGAAAAATGAAAACCC
ACCACGAAAGTAGCTTTAACCACACCTGAACTCATGACAACTAAGAAA
>JS20-1_9-2-MiFish_Sponge
CACCGCGGTTATACGAGAGGCCCAAGTTGATAGGCACCGGCGTAAAGAGTGGTTAGGGCA
CATAAACTCCAACTAAAGCCGAACGCCCCCAGAGCTGTTATACGCACCCGAAGGTATGAA
GAACCACCACGAAAGTGGCTTTATTCCCCCCGAACCCACGAAAGCTATGCCA
>JS20-1_12-1-MiFish_Sponge
CACCGCGGTTATACGAGAGGCCCAAGTTGATAGGCACCGGCGTAAAGAGTGGTTAGGGCA
CATAAACTCCAACTAAAGCCGAACGCCCCCAGAGCTGTTATACGCACCCGAAGGTATGAA
GAACCACCACGAAAGTGGCTTTATTCCCCCCGAACCCACGAAAGCTATGCCA
>JS20-2W6_Seawater
CACCGCGGTTATACGAGAGGCCCAAGTTGATAGGCACCGGCGTAAAGAGTGGTTAGGGCA
CATAAACTCCAACTAAAGCCGAACGCCCCCAGAGCTGTTATACGCACCCGAAGGTATGAA
GAACCACCACGAAAGTGGCTTTATTCCCCCCGAACCCACGAAAGCTATGCCA
>HK20-3WS2_Seawater
CACCGCGGTTATACGAGAGGCCCAAGTTGATGAAAACGGCGTAAAGAGTGGTTAAGAAAA
TAAATAAAATAAGGCCGAATAGTCTCATTGCAGTTATACGTTTACGAGACCACTAAGCAC
ATTCACGAAAGTAGCCTTAGCAGAATTCTGACTCCACGAGAGCCATAAGA
>JS20-2W4-1_Seawater
CACCGCGGTTATACGAGAGGCCCAAGTTGATGAAAACGGCGTAAAGAGTGGTTAAGAAAA
```

```
TAAATAAAATAAGGCCGAATAGTCTCATTGCAGTTATACGTTTACGAGACCACTAAGCAC
ATTCACGAAAGTAGCCTTAGCAGAATTCTGACTCCACGAGAGCCATAAGA
>JS20-1_10-2-MiFish_Sponge
CACCGCGGTTATACGAGAGGCCCAAGTTGATGAAAACGGCGTAAAGAGTGGTTAAGAAAA
TAAATAAAATAAGGCCGAATAGTCTCATTGCAGTTATACGTTTACGAGACCACTAAGCAC
ATTCACGAAAGTAGCCTTAGCAGAATTCTGACTCCACGAGAGCCATAAGA
>JS20-2W3_Seawater
CACCGCGGTTATACGAGAGGCTCAAGTTGAGAGAACTACGGCGTAAAGGGTGGTTAAGGT
AAACTTAAAAAATAAAGCCGAACCCCCTCAACACTGTAATACGCCCCCGAGGGGAGGAAG
CCCAATCACGAAAGTGGCTTTACAACTCCTGAACCCACGAAAGCTAGGGCA
>JS20-1_10-4-MiFish_Sponge
CACCGCGGTTATACGAGAGGCTCAAGTTGAGAGAACTACGGCGTAAAGGGTGGTTAAGGT
AAACTTAAAAAATAAAGCCGAACCCCCTCAACACTGTAATACGCCCCCGGGGGGAGGAAG
CCCAATCACGAAAGTGGCTTTACAACTCCTGAACCCACGAAAGCTAGGGCA
```
