# Supplementary material for: Utilizing environmental DNA and imaging to study the deep-sea fish community of Takuyo-Daigo Seamount
Source: NPJ Biodivers. 2024 May 31;3:14. doi: 10.1038/s44185-024-00042-w (PMC11331990; doi:10.1038/s44185-024-00042-w)
Supplement: Supplementary file 3 — Supplementary Figures [file 44185_2024_42_MOESM3_ESM.pdf]

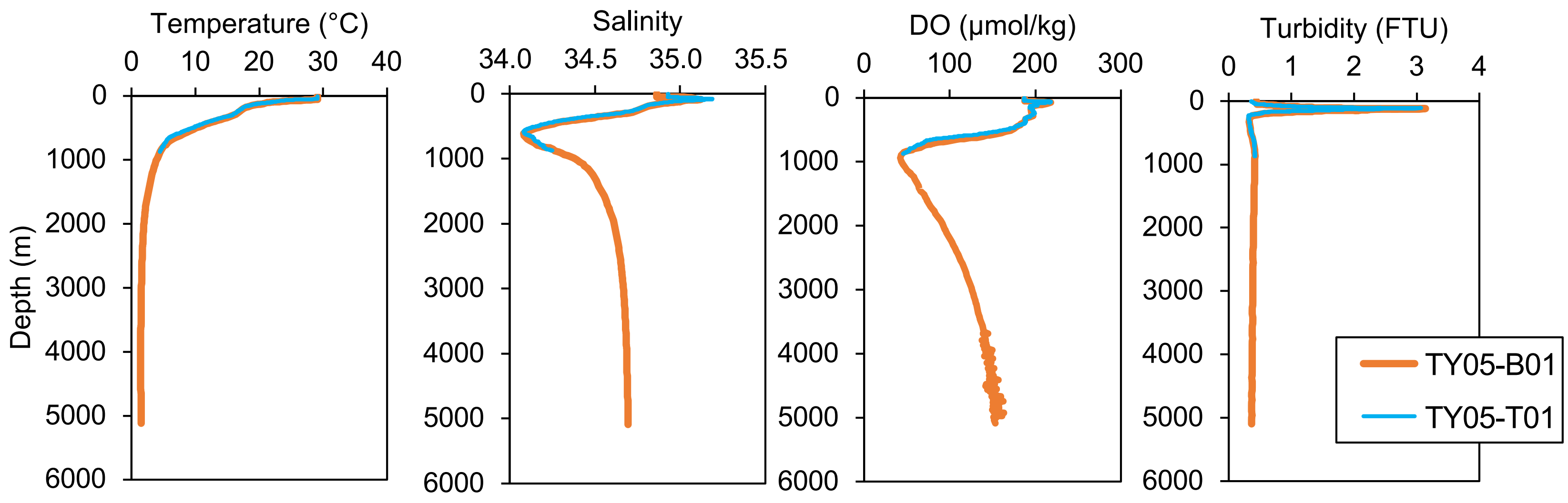

Supplementary Figure 1 Iguchi et al.

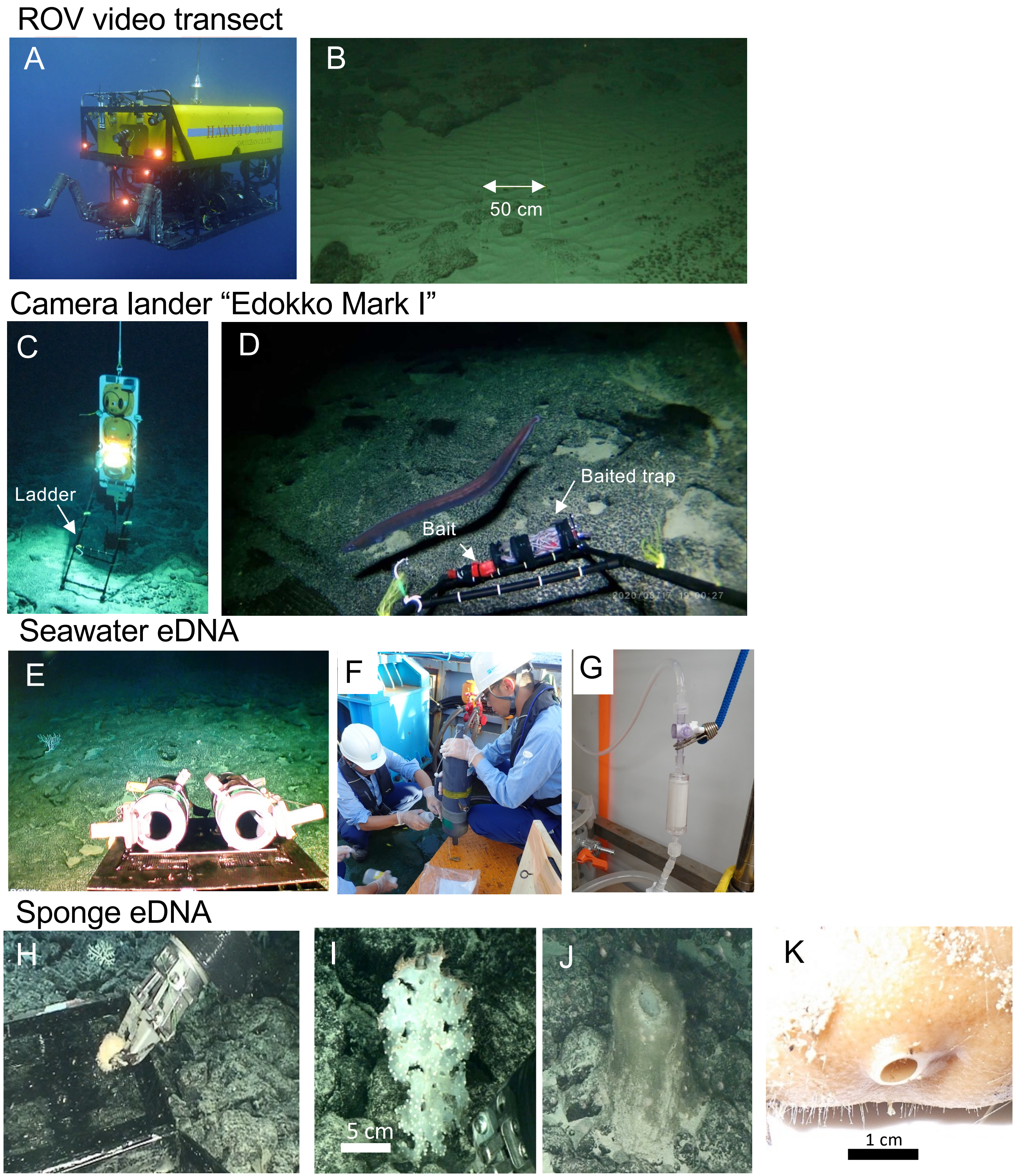

Supplementary Figure 2
Iguchi et al.

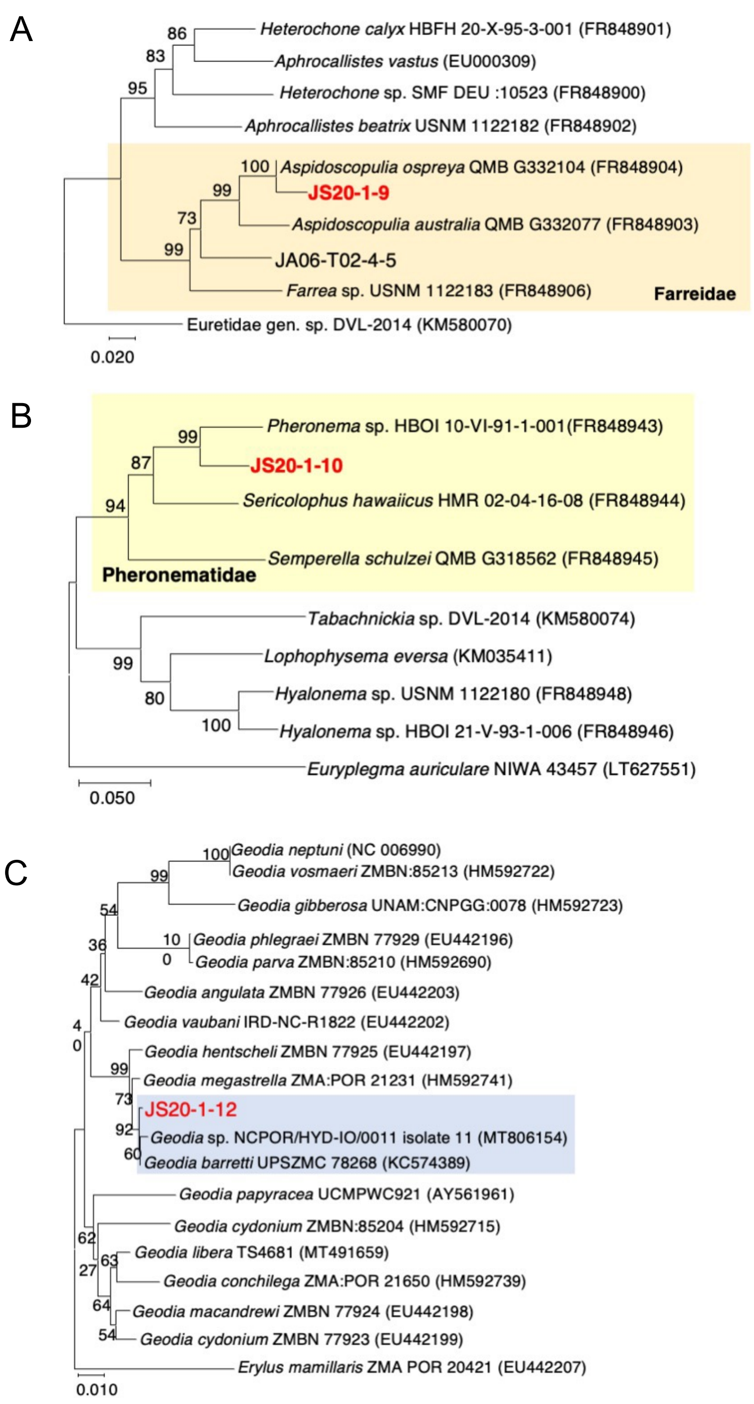

Supplementary Figure 3 Iguchi et al.

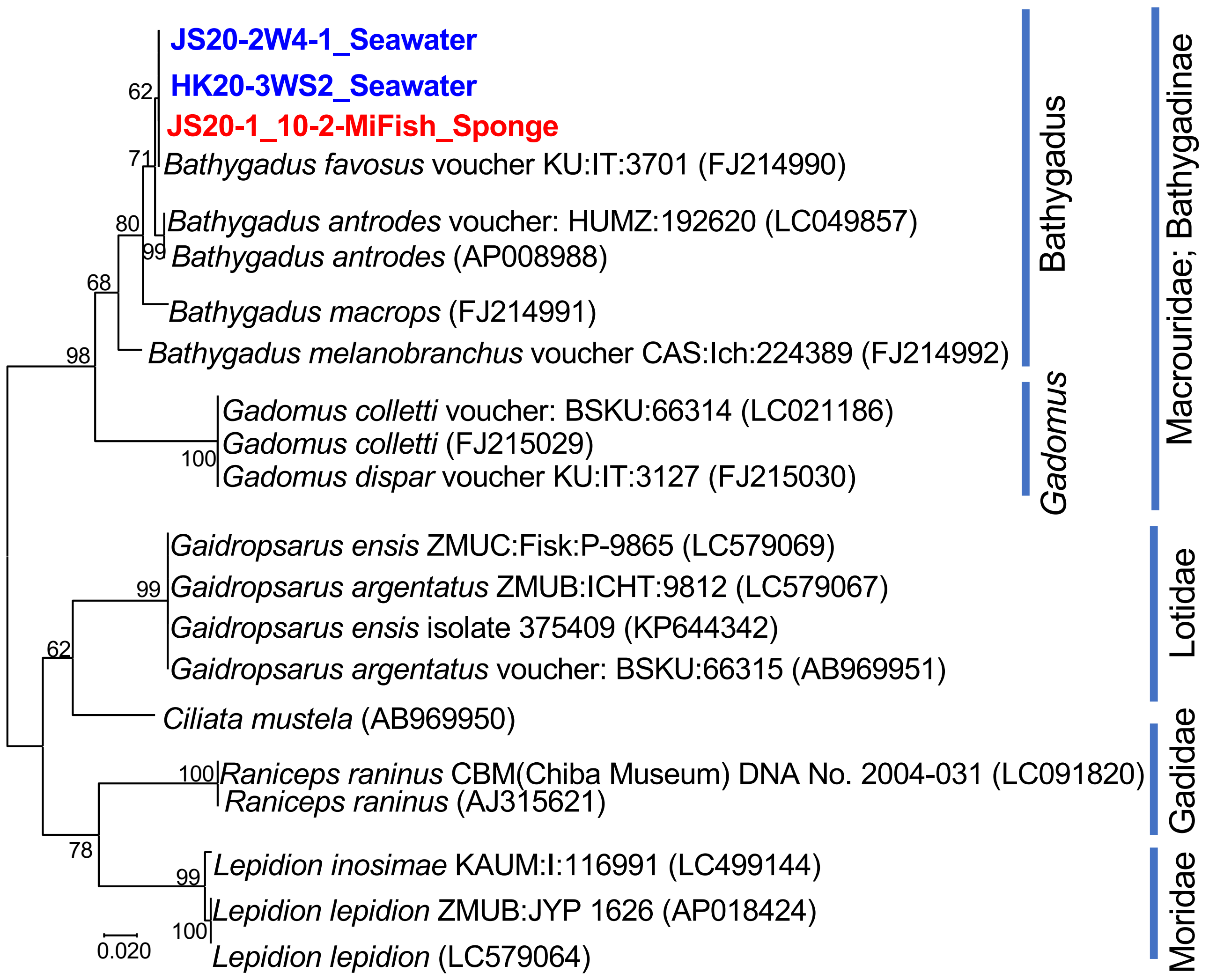

Supplementary Figure 4 Iguchi et al.

分岐図の線の太さを調整

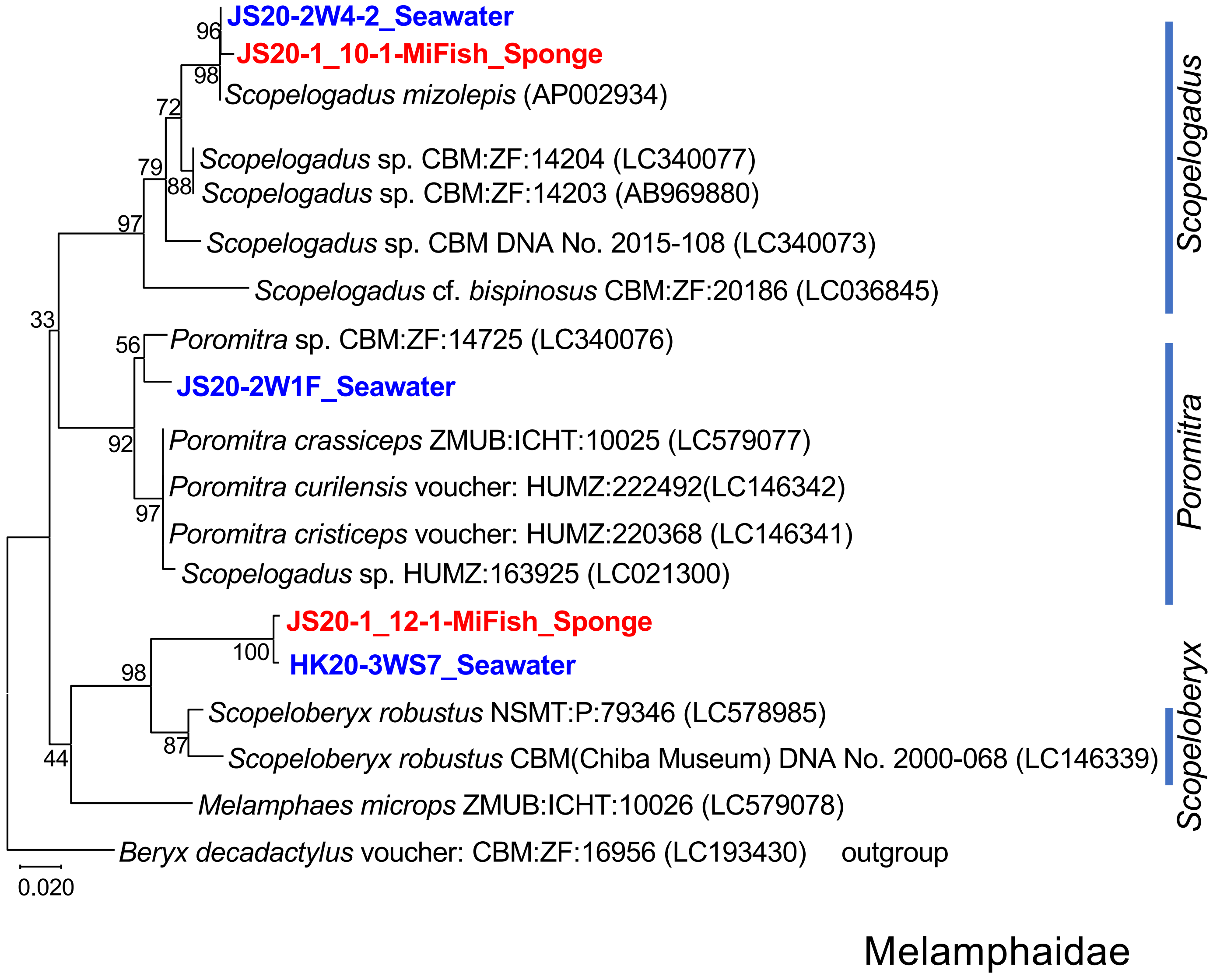

Supplementary Figure 5 Iguchi et al.

分岐図の線の太さを調整

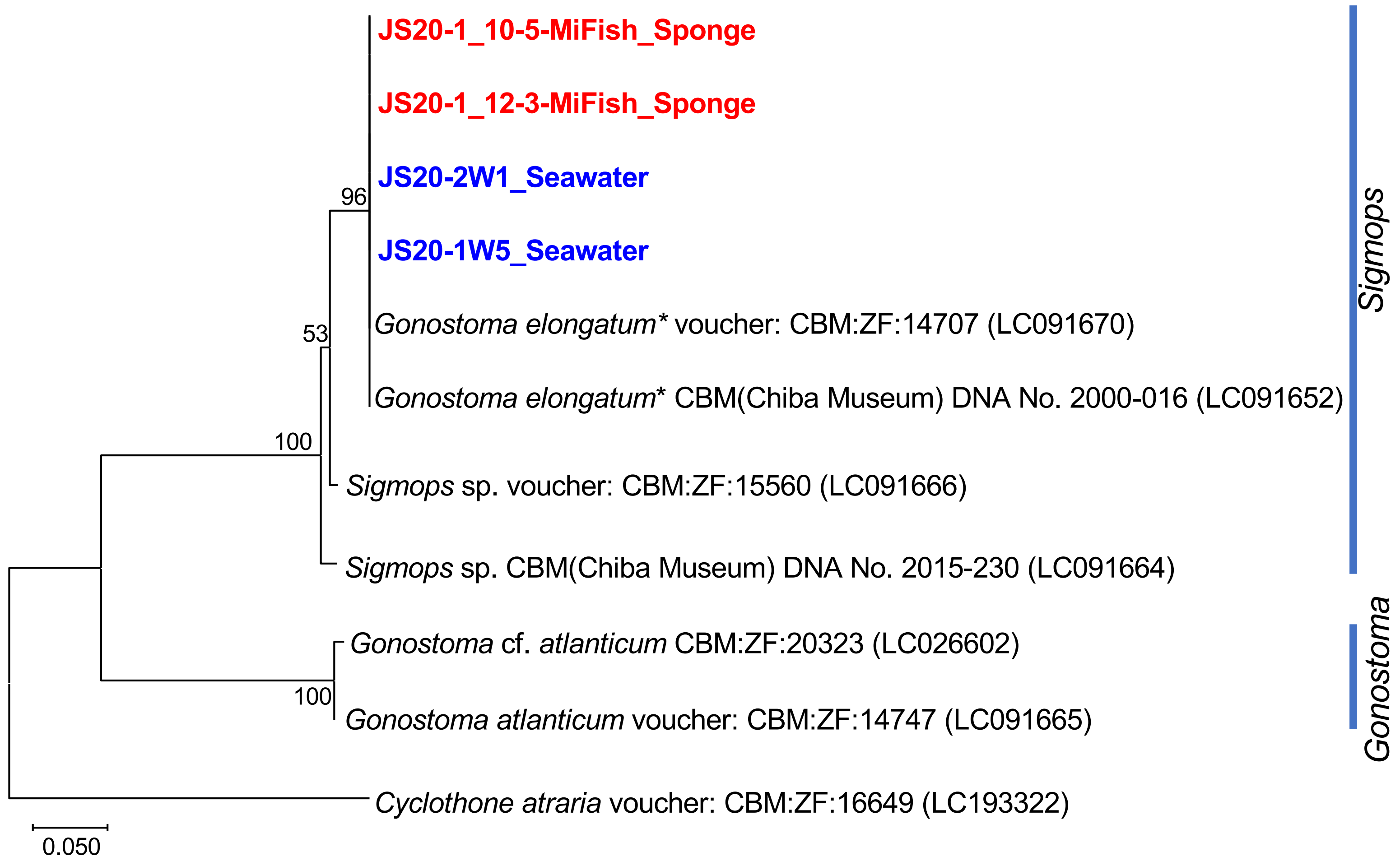

Gonostomatidae

**Gonostoma elongatum* is a synonym of *Sigmops elongatus*

Supplementary Figure 6 Iguchi et al.

分岐図の線の太さを調整

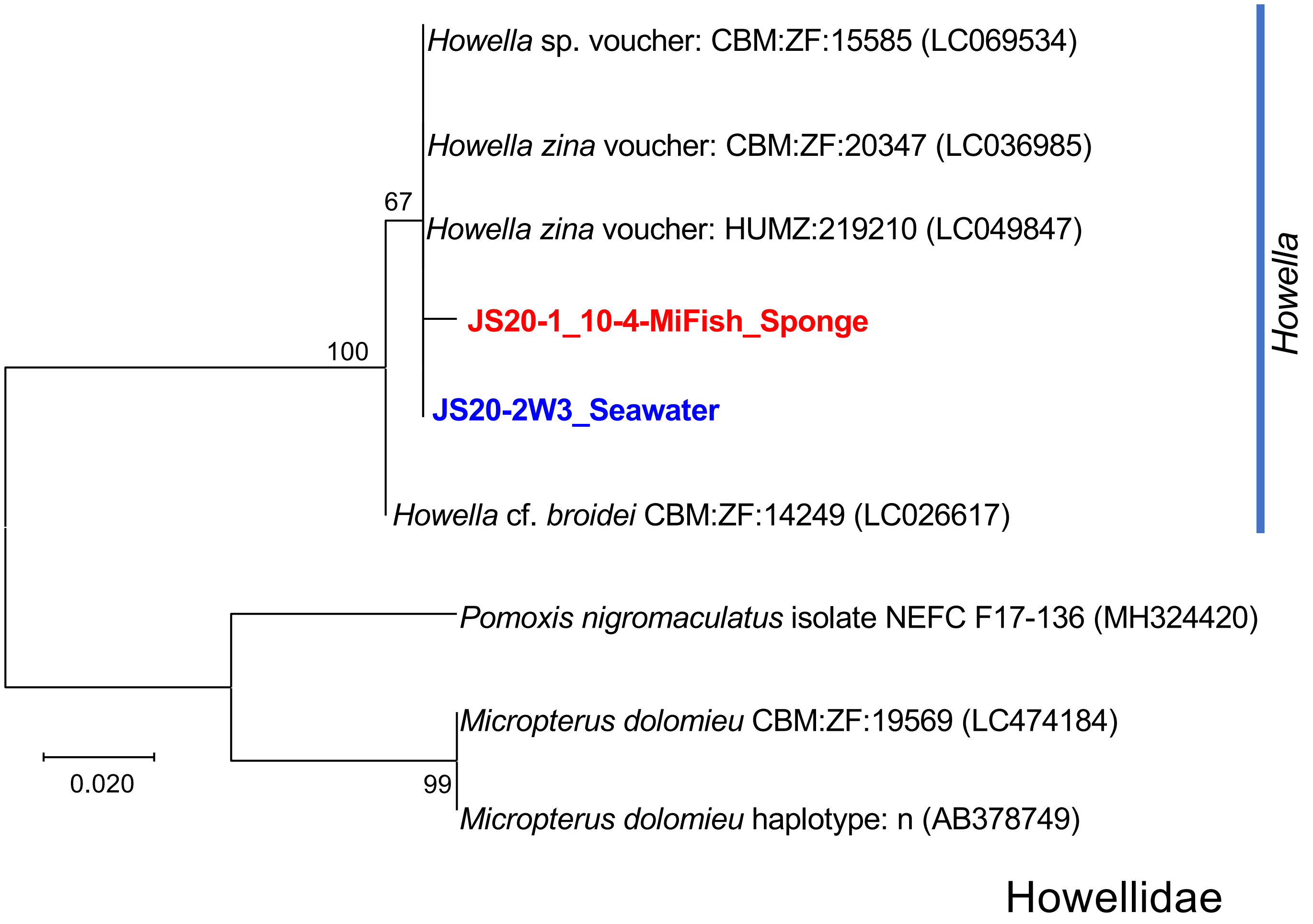

Supplementary Figure 7 Iguchi et al.

分岐図の線の太さを調整

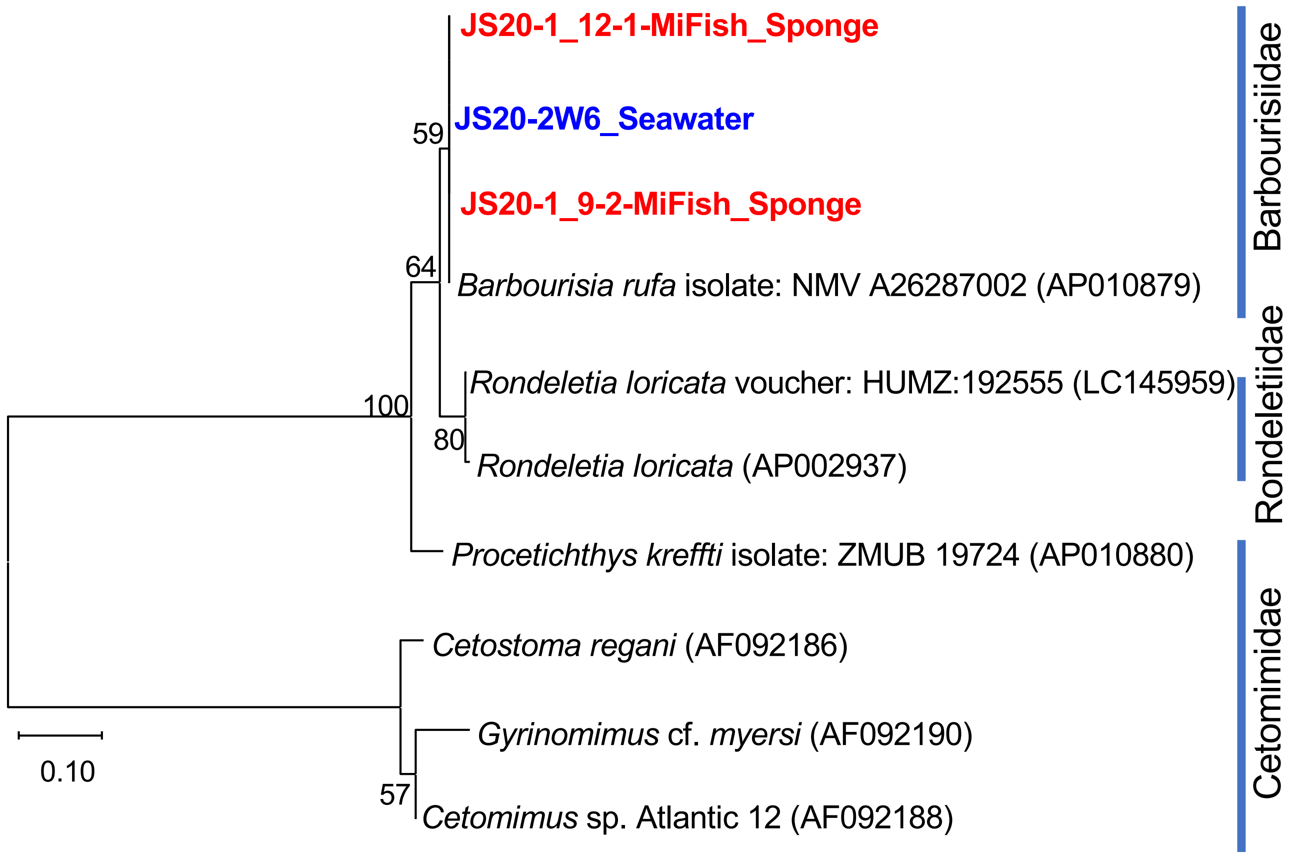

Fig. S8

分岐図の線の太さを調整

Supplementary Figure 8 Iguchi et al.

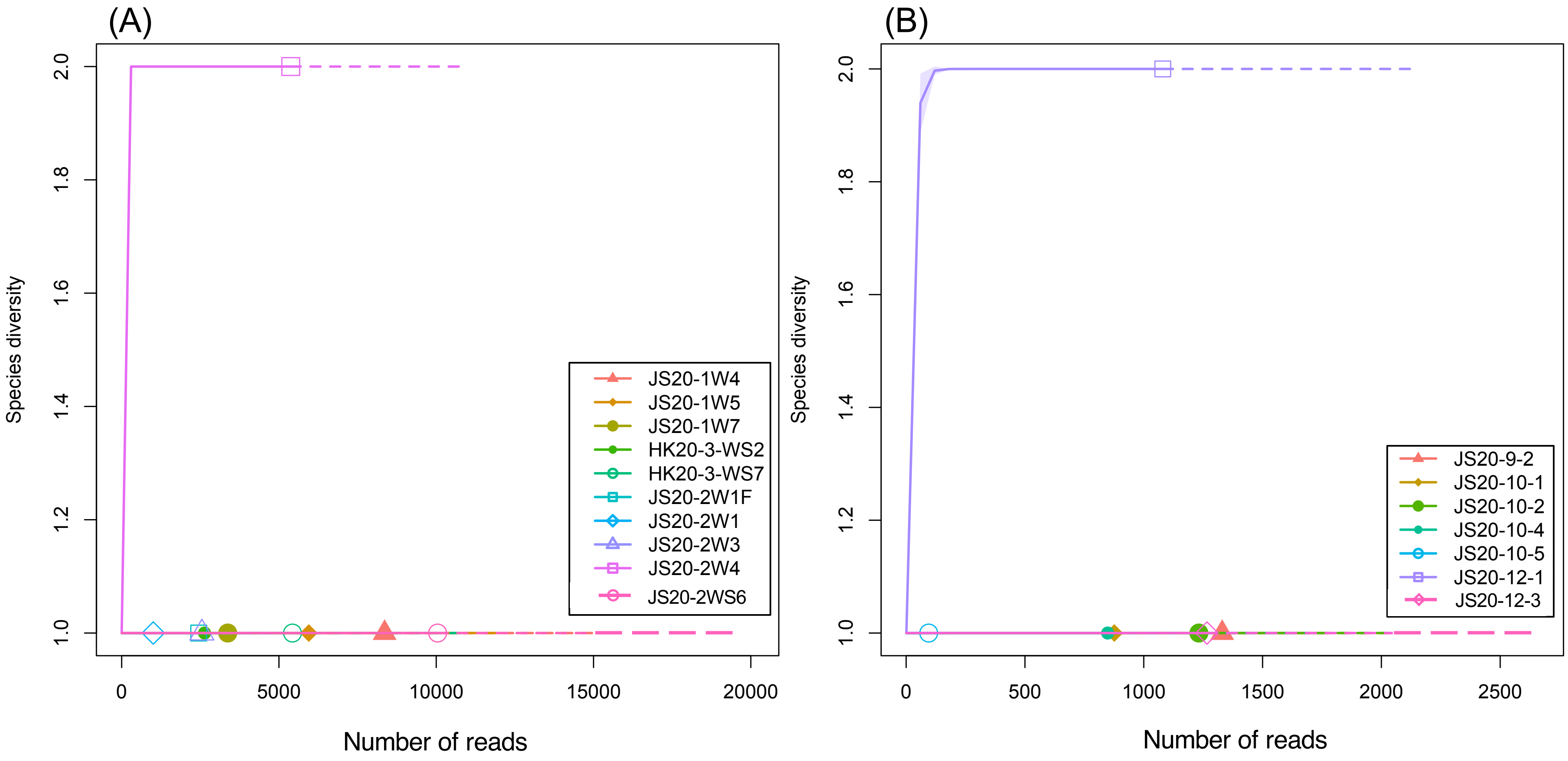

Supplementary Figure 9 Iguchi et al.

分岐図の線の太さを調整
